# Proteases as antimalarial targets: strategies for genetic, chemical, and therapeutic validation

**DOI:** 10.1111/febs.14130

**Published:** 2017-07-03

**Authors:** Edgar Deu

**Affiliations:** ^1^ Chemical Biology Approaches to Malaria Laboratory The Francis Crick Institute London UK

**Keywords:** malaria, protease, target validation

## Abstract

Malaria is a devastating parasitic disease affecting half of the world's population. The rapid emergence of resistance against new antimalarial drugs, including artemisinin‐based therapies, has made the development of drugs with novel mechanisms of action extremely urgent. Proteases are enzymes proven to be well suited for target‐based drug development due to our knowledge of their enzymatic mechanisms and active site structures. More importantly, *Plasmodium* proteases have been shown to be involved in a variety of pathways that are essential for parasite survival. However, pharmacological rather than target‐based approaches have dominated the field of antimalarial drug development, in part due to the challenge of robustly validating *Plasmodium* targets at the genetic level. Fortunately, over the last few years there has been significant progress in the development of efficient genetic methods to modify the parasite, including several conditional approaches. This progress is finally allowing us not only to validate essential genes genetically, but also to study their molecular functions. In this review, I present our current understanding of the biological role proteases play in the malaria parasite life cycle. I also discuss how the recent advances in *Plasmodium* genetics, the improvement of protease‐oriented chemical biology approaches, and the development of malaria‐focused pharmacological assays, can be combined to achieve a robust biological, chemical and therapeutic validation of *Plasmodium* proteases as viable drug targets.

**Abbreviations**
ABP: activity‐based probe
ACT: artemisinin‐based combination therapy
A‐M17: M17‐family leucyl aminopeptidase
A‐M18: M18‐family aspartyl aminopeptidase
A‐M1: M1‐family alanyl aminopeptidase
AMA1: apical membrane antigen 1
APP: aminopeptidase P
ATG8: autophagy 8
BP: bergheipain
CETSA: cellular thermal shift assay
cKD: conditional knock down
cKO: conditional knock out
ClpP: caseinolytic protease
COFRADIC: combined fractional diagonal chromatography
CoSeSuL: counter selection substrate libraries
CRISPR: clustered regulatory interspaced short palindromic repeats
DART: drug affinity response target stability
DiCre: dimerizable Cre recombinase
DPAP: dipeptidyl aminopeptidase
DUB: deubiquitylating enzyme
EMP2: erythrocyte membrane protein 2
ER: endoplasmic reticulum
EXP: exported protein 1
FP: falcipain
FtsH: filamentous temperature‐sensitive H peptidase
GBP130: glycophorin‐binding protein 130
HslV: heat‐shock locus V
Hsp70: heat‐shock protein 70
HUB1: homology to ubiquitin 1
HyCoSuL: hybrid combinatorial substrate libraries
iRBC: infected red blood cell
MetAP: methionine aminopeptidase
MIP: mitochondrial intermediate peptidase
MPP: mitochondrial processing peptidase
MSP: merozoite surface protein
NEDD8: Neural Precursor Cell Expressed Developmentally Downregulated 8
PARL: presenilins‐associated rhomboid‐like protein
PEXEL: protein export element
PM: plasmepsin
PTEX: *Plasmodium* translocon for exported proteins
PTRAMP: thrombospondin‐related apical membrane protein
PVM: parasitophorous vacuole membrane
PV: parasitophorous vacuole
RAMA: rhoptry‐associated membrane antigen
RAP1: rhoptry‐associated protein 1
RBCM: red blood cell membrane
RBC: red blood cell
RhopH3: high molecular weight rhoptry protein 3
ROM: rhomboid
S2P: site‐2 protease
SAR: structure–activity relationship
SCID: severe combined immunodeficiency
SENP: sentrin‐specific peptidase
SERA: serine repeat antigen
SNP: single‐nucleotide polymorphism
SPP: aspartyl signal peptide peptidase
SP: serine signal peptidase
SUB: subtilisin‐like protease
SUMO: small ubiquitin‐like modifier
TAILS: terminal amine isotopic labelling of substrates
TPP: thermal proteome profiling
TRAP: thrombospondin‐related anonymous protein
UBP1: ubiquitin peptidase 1
UCH: ubiquitin C‐terminal hydrolase
URM1: ubiquitin‐related modifier 1
USP14: ubiquitin‐specific peptidase 14
WT: wild‐type

## Introduction

Over the last decade, the world has seen a significant decrease in malaria incidence, from 1 to 2 million deaths in 2000 to an estimate of half a million this year 1. This is mainly due to the global distribution of insecticide‐impregnated bed nets and the introduction of artemisinin‐based combination therapy (ACT) as the recommended antimalarial treatment. Unfortunately, mosquitoes are becoming increasingly resistant to insecticides 2, and artemisinin resistance is rapidly emerging 3. Given that most antimalarial drug development programs currently in clinical trials rely on artemisinin analogues and ACTs 4, it is crucial to develop drugs with novel mechanisms of action in order to stay ahead in our fight against drug resistance.

**Figure 1:**
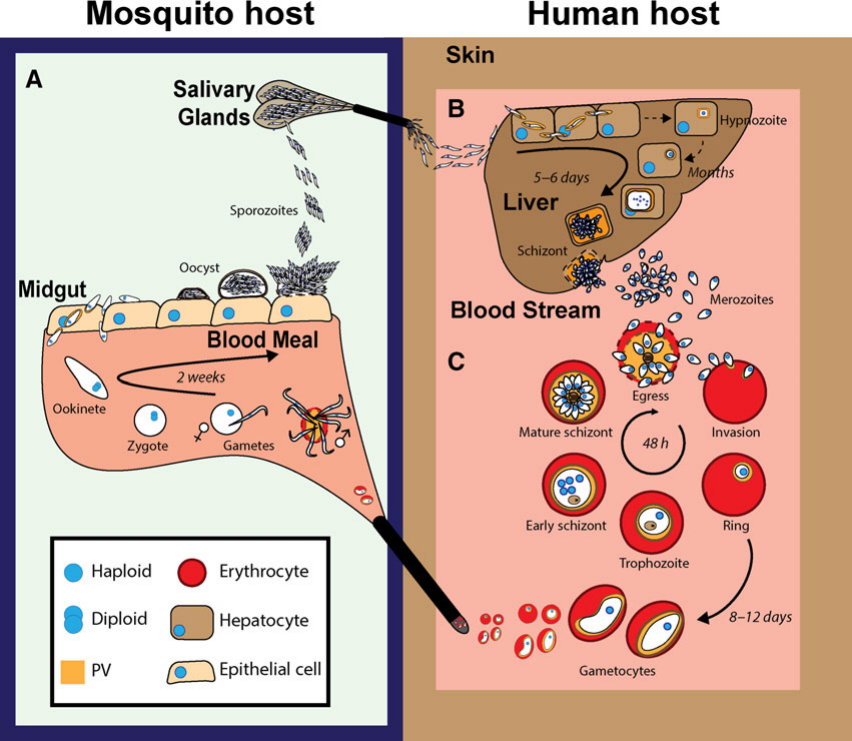
The malaria parasite life cycle. Schematic representation of the insect (A), liver (B) and blood (C) stages of parasite development. The timing of parasite development at each stage is indicated for *Plasmodium falciparum*. Note that gametocyte development is much faster in other *Plasmodium* spp., and that *P. falciparum* does not form hypnozoites.

Malaria infection takes place during a mosquito bite when infected female *Anopheles* mosquitoes inject highly motile parasites (sporozoites) into the skin (Fig. 1A). Sporozoites traverse the skin barrier, reach the blood stream, and travel to the liver where they establish an asymptomatic infection in hepatocytes (Fig. 1B). There they multiply asexually to form thousands of infective merozoites that are released into the blood stream, thus starting the ~ 48 h erythrocytic cycle (Fig. 1C). Merozoites actively invade red blood cells (RBCs) using an actin/myosin motor. Invagination of the RBC membrane during invasion contributes to the formation of the parasitophorous vacuole, a compartment within which the parasite develops isolated from the RBC cytosol. After RBC invasion, the asexual developmental cycle is initiated. Morphologically defined ‘ring stage’ parasites mature and grow within the RBC as they degrade the host haemoglobin (described as the trophozoite stage). Multiple rounds of asynchronous nuclear division occur during the process of schizogony (schizont stage), followed by a concerted invagination of the plasma membrane, which produces 20–32 daughter merozoites. Once matured, merozoites egress from the infected RBCs (iRBCs) and invade new erythrocytes, thus restarting the cycle (Fig. 1C). Some blood‐circulating parasites develop into male and female gametocytes, which can be taken up by another mosquito during a blood meal. These mature into male and female gametes within the mosquito midgut and fuse to form a zygote, which then develops into a diploid ookinete. This motile parasite form traverses the midgut wall and forms an oocyst within which parasites multiply asexually to form thousands of haploid sporozoites. After egress, sporozoites travel to the mosquito salivary glands, from where they are transmitted to the next human host (Fig. 1A).

The synchronous release of parasites and toxic material from the iRBC during the erythrocytic stages is responsible for the cyclic symptoms of the disease including fever, chills, nausea, body aches and headaches, which can lead to serious complications such as severe anaemia, acute respiratory syndrome, hypoglycaemia, metabolic acidosis, haemoglobinuria, acute kidney failure or cerebral malarial. An antimalarial drug should therefore primarily target the erythrocytic stages and, if possible, also the liver and/or sexual stages to prevent transmission. Proteases are one of the preferred enzyme families for target‐based drug development due to their role in a variety of human diseases and their well‐characterised catalytic mechanisms and active site structures. Indeed, protease inhibitors are currently being used to treat cancer, diabetes, hypertension, myocardial infarction, acute lung injury, hepatitis C and AIDS 5, 6. Based on the MEROPS protease database, *Plasmodium* genomes encode around 170 predicted proteases. However, only one‐third of these proteases have been studied, and among those, very few have been thoroughly characterised or validated as drug targets (Table 1). That said, proteases perform a variety of crucial biological functions at all stages of parasite development, and some of them are likely to be ideal therapeutic targets 7.

**Table 1:** Studied *Plasmodium* proteases: biological functions and target validation

Family	Protease name	Gene ID PF3D7_#	Biological function	Localisation	Chemical validation	Genetic validation^a^	Confirmed protease activity
Aspartate	PM‐I	1407900	Haemoglobin degradation	FV	Yes 77	Redundant 75, 76	Yes 72
	PM‐II	1408000	Haemoglobin degradation	FV			
	PM‐III (HAP)	1408100	Haemoglobin degradation	FV			
	PM‐IV	1407800	Haemoglobin degradation	FV			
	PM‐V	1323500	Protein export	ER	Yes 122, 123	Essential 60, 61	Yes 60
	PM‐VI	0311700	Sporozoite formation		No	Important for transmission 54	No
	PM‐VII	1033800	*Midgut transversal*		No	Redundant 53	No
	PM‐VIII	1465700	Sporozoites motility		No	Redundant in RBCs Essential for transmission^*Pb*^ 55	
	PM‐IX	1430200	Invasion		Yes^b^	Essential^b^	Yes^b^
	PM‐X	0808200	*Midgut transversal*		No	ND 52	No
	SPP	1457000	Protein traffic	ER	Yes 21, 90	Likely important 90	Yes
Cysteine	FP1	1458000	Maybe invasion	*Cytosol*	Partial 56	Redundant 57	Partial (ABP) 56
	FP2a	1115700	Haemoglobin degradation	FV	Yes 74	Redundant 74	Yes 67
	FP2b	1115300	Haemoglobin degradation	FV		Redundant 74	Yes 68
	FP3	1115400	Haemoglobin degradation	FV		Redundant^b^	Yes 69
	DPAP1	1116700	Haemoglobin degradation	FV	Partial 22	Likely important 79	Yes 80
	DPAP2	1247800	Gametocyte egress	Osmiophilic bodies	No	Important 37	Partial (ABP) 36
	DPAP3	0404700	Invasion	Apical organelle	No	Important (cKO^b^)	Partial (ABP) 35
	SERA6	0207500	Egress	PV	No	Essential 29, 31	Auto‐processing^*Pb*^ 137
	SERA7	0207400	Egress	PV	No	Redundant 29	No
	SERA8	0207300	Egress Sporozoite egress	PV	No	Redundant in RBC 29 Likely essential for transmission^*Pb*^ 30	No
	Metacaspase‐1	1354800	Cell death	Cytosol	No	Redundant^*Pb*^ 112	Yes 110
	Calpain‐1	1362400	Nuclear division	Cytosol & Nucleus	No	Important 108	No
	UCHL3	1460400	N.D.	N.D.	No	No	Partial (ABP) 98
	UCH54	1117100	N.D.	N.D.	No	No	Partial (ABP) 99
	UBP1	0104300	Artemisinin resistance 100	N.D.	No	No	No
	USP14	0527200	Protein homeostasis	Cytosol	Partial 101	No	Yes 101
	SENP1	1233900	Invasion	*Cytosol*	Partial 18	No	Yes 18
Metallo	Falcilysin	1360800	Haemoglobin degradation Api transit peptide degradation	Digestive Vacuole/Apicoplast	No	Likely important 78	Yes 70
	Stromal processing peptidase	1440200	Process apicoplast transit peptide	*Apicoplast* 92	No	No	No
	PfA‐M1	1311800	Protein catabolism	FV/cytoplasm	Yes 83, 84, 85, 86	Likely important 81	Yes 81
	PfA‐M17/LAP	1446200	Protein catabolism	Cytoplasm	Yes 83, 84, 85, 86	Likely important 81	Yes 81
	PfA‐M18/DAP	0932300	Protein catabolism	Cytoplasm	No	Redundant 81	Yes 82
	Pf‐APP	1454400	Protein catabolism	FV/cytoplasm	No	Likely important 81	Yes 81
	PfMetAP1a	0527300	Removal Nt Met	*Mitochondria*	No	No	Yes 96
	PfMetAP1b	1015300	Removal Nt Met	*Cytosol*	Yes 96	No	Yes 96
	PfMetAP1c	0804400	Removal Nt Met	*Apicoplast*	No	No	Yes 96
	PfMetAP2	1344600	Removal Nt Met	*Apicoplast*	Partial 94, 95	No	Partial 94
	PfFtsH1	1133400	Mitochondria protein quality control	Mitochondria	No	No	Yes 105
	S2P	1305600	Protein quality control	Likely Golgi	No	Important ^*Pb*^ 102	No
Serine	SUB1	0507500	Egress/Invasion Merozoite formation and egress in hepatocytes^*Pb*^	Exonemes to PV	Yes 28, 35	Essential (cKO^b^) Essential in liver stages^*Pb*^ 33, 34	Yes 28
	SUB2	1136900	Invasion	Micronemes to Plasma Membrane Osmiophilic bodies	No	Essential (cKO^b^) 49	Yes 49
	SUB3	0507200	N.D.	N.D.	No	Redundant 28	No
	ROM1	1114100	RBC invasion Hepatocyte invasion^*Pb, Py*^	Apical organelle	No	Likely important 43, 46, 47, 48	Yes 41
	ROM3	0828000	Sporozoite development	N.D.	No	Essential 48	No
	ROM4	0506900	Invasion	Merozoite surface	No	Likely important 41, 48 ^*Pb*^	Yes 41
	ROM6	1345200	*Mitchondria protein quality control*	*Mitochondria*	No	Likely important^*Pb*^ 48	No
	ROM7	1358300	*Apicoplast protein quality control*	*Apicoplast*	No	Likely important^*Pb*^ 48	No
	ROM8	1411200	N.D.	N.D.	No	Likely important^*Pb*^ 48	No
	ROM9	0515100	N.D.	N.D.	No	Redundant^*Pb*^ 48	No
	ROM10	0618600	N.D.	N.D.	No	Redundant^*Pb*^ 48	No
	ClpAP	0307400	Apicoplast biogenesis/maintenance	Apicoplast	Yes 106	No	Yes 106
	PAP	1401300	RBC deformability/cytoadhesion	RBC cytosol	No	Likely important 65	Yes 65
	SP18	1320400	Cleavage signal peptide	*ER*	No	No	Yes 88
	SP21	1331300	Cleavage signal peptide	ER	No	No	Yes 89
Threonine	Proteasome β1 β2 β5	0518300 1470900 1011400	Protein homeostasis	Cytosol	Yes 20	Essential	Yes 25
	PfClpQ/HslV	1230400	Mitochondria biogenesis and function	Mitochondria	No	Essential 104	Yes 104

Italic font indicates predicted localisation or function. Protease function, localisation and validation refer to *Plasmodium falciparum* genes unless indicated with ^*Pb/Py*^ for *Plasmodium berghei/yoelii*. ^a^ Definition for genetic validation: Likely important: KO attempts have been unsuccessful; Essential: cKO or cKD approach used to prove essentiality of a gene; Important: protease KO or KD shows a significant fitness cost; Redundant: gene KO has no pronounced phenotype. ^b^ Unpublished (D. Soldati‐Favre, D. E. Goldberg & M. J. Blackman, personal communication).

Target‐based approaches have so far not been very successful in developing antimalarial drugs, in part due to the difficulty of genetically validating targets in *Plasmodium* spp. Moreover, early protease‐targeting drug development efforts focused on inhibiting proteases involved in the degradation of haemoglobin (falcipains and plasmepsins). These programs lost some of their momentum once advances in malaria genetics showed a high level of proteolytic redundancy in this pathway 8. Although robust validation of antimalarial targets has been difficult in the past, the advent of new, faster, and broadly applicable genetic methods 9, the recent implementation of malaria‐specific pharmacological and phenotypic assays 10, the use of new *in vivo* malaria models 11, 12, 13, 14, and the increased involvement of the pharmaceutical industry in public–private partnerships 15, 16, provide a unique opportunity to determine which *Plasmodium* proteases are likely to be viable antimalarial targets.

In this review, I provide an overview of the role of proteases in *Plasmodium* biology with a strong emphasis on *Plasmodium falciparum*, being the most virulent and best studied *Plasmodium* species. I then present my opinion about the need to validate antimalarial targets at the genetic, biological, chemical and therapeutic levels before embarking on long and expensive drug development campaigns. In particular, I emphasise how the combination of conditional genetic methods, malaria‐specific pharmacological assays, and chemical biology approaches can be used to achieve robust target validation.

## Role of *Plasmodium* proteases in parasite biology

*Plasmodium* proteases play both regulatory and effector roles in a variety of essential biological processes. However, it is important to consider which among the ~ 170 predicted *Plasmodium* proteases are likely to be viable therapeutic targets. *A priori*, proteases that are not conserved in humans are more likely to have parasite‐specific functions, and their inhibitors might be less prone to inactivate host proteases. That said, the following points should be carefully considered before discarding *Plasmodium* proteases that have human homologues:

1. Phylogenetic conservation does not always translate into structural conservation of the active site. Indeed, significant differences in substrate and/or inhibitor specificity between *Plasmodium* and human proteases have been documented 17, 18, 19 and can be taken advantage of to develop *Plasmodium*‐specific inhibitors.
2. Off‐target inhibition of host proteases does not always need to lead to adverse effects. For example, potent *Plasmodium* proteasome inhibitors can have good antiparasitic activity with minimal toxicity if they do not inhibit the host β2 subunit; inhibition of the host β5 subunit is well tolerated 20. Similarly, potent ER signal peptide peptidase (SPP) 21 or dipeptidyl aminopeptidase (DPAP) inhibitors 22 with low nanomolar antiparasitic activity are not toxic to host cells despite inhibiting their corresponding human homologues. Finally, the vinyl sulfone inhibitor K117777, a potent cruzain inhibitor in preclinical trials for the treatment of Chagas disease, has a very safe toxicity profile despite clear evidence that it also targets multiple host cysteine cathepsins 23. It is also likely that inhibition of many human proteases during the short course of treatment for acute malaria (1–3 days) will not result in adverse effects.
3. Targeting host proteases might be beneficial. Most malaria symptoms result from a pronounced dysregulation of the immune and inflammatory responses during the parasite erythrocytic cycle. Given the central role of proteases in these processes, it is important to consider whether off‐target inhibition of certain human proteases might not result in beneficial adjuvant effects. Unfortunately, the role of host proteases in malaria pathology is poorly understood. In addition, there are a few examples showing that erythrocytic enzymes play a role in parasite development. For example, human calpain‐1 is thought to be activated at the time of egress to assist parasites escape the iRBC 24. One theoretical advantage of targeting human proteases to fight infectious diseases is that the pathogen is less likely to acquire resistance to the drug.
4. Repurposing compounds from industry is a cost‐effective strategy to tackle neglected diseases. Because pharma industry efforts are mainly focused on targeting noninfectious human disorders (cancer, diabetes, autoimmune and neurological diseases, etc.), most protease‐oriented drug development programs target human enzymes. Focused libraries of inhibitors can therefore be repurposed to develop potent inhibitors against *Plasmodium* homologues relatively quickly. This also encourages successful collaborations with industry and provides access to valuable medicinal chemistry, pharmacological and structural biology knowledge. For example, the K11777 inhibitor mentioned above was developed by Khepri Pharmaceuticals as a cathepsin S inhibitor. Also, the work that led to the development of *Plasmodium*‐specific proteasome inhibitors 20 originated from a screen of proteasome inhibitors that was synthesised by Proteolyx 25 during the development of carfilzomib for the treatment of multiple myeloma.

Another important point to consider is whether a protease performing a parasite‐specific function is a better target than one performing a function conserved in eukaryotes. Proteases play important roles in a variety of biological processes such as protein homeostasis, trafficking, cell signalling, catabolism or cell death, and most of these are also conserved in apicomplexan parasites. However, *Plasmodium* spp. have also evolved parasite‐specific proteolytic pathways allowing them to replicate within host cells and evade the immune system efficiently. Parasite‐specific proteolytic functions include mechanisms to get in and out of the RBC, pathways to degrade haemoglobin, and mechanisms to modify the erythrocyte cytosol and membrane to acquire nutrients and evade the immune system. Although proteases involved in these key biological processes are potential drug targets, their activities might only be required for a short period of time during the parasite life cycle. On the other hand, proteases that play core biological functions, such as protein homeostasis or protein traffic, are more likely to be essential at all stages of parasite development.

### *Plasmodium*‐specific proteolytic pathways

#### Getting in and out of the host cell

**Figure 2:**
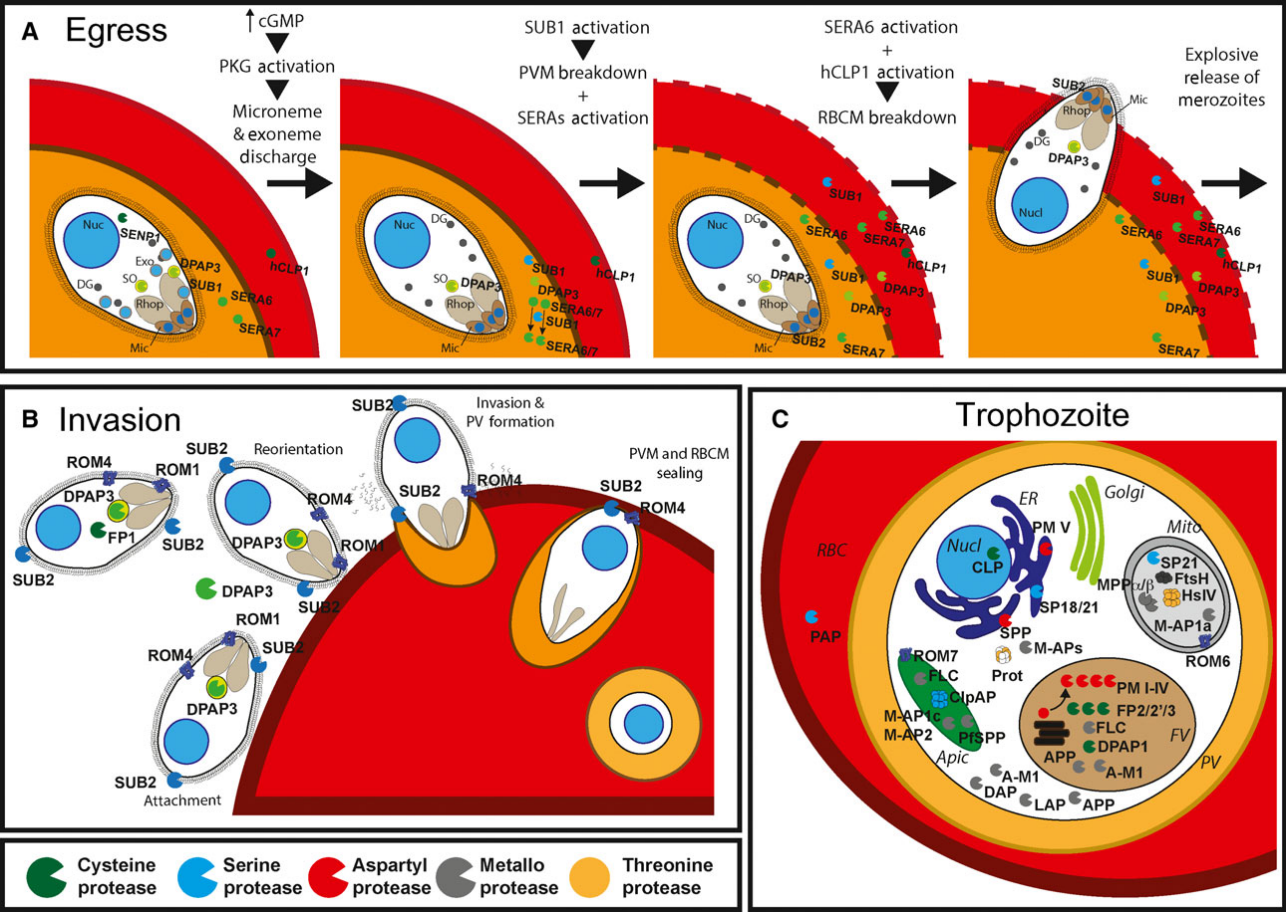
Role of proteases during the erythrocytic cycle. (A) Role of proteases in parasite egress. (B) Role of proteases in RBC invasion. (C) Core biological functions of malaria proteases illustrated at trophozoite stage. Circles indicate zymogen/inactive protease forms, pacman shapes indicate active proteases. Asp, Cys, Ser, Thr and metalloproteases are shown in red, green, blue, orange and grey, respectively. Nucl, nucleus; Exo, exonemes; Mic, micronemes; Rhop, rhoptries; PV, parasitophorous vacuole; ER, endoplasmic reticulum; Mito, mitochondria; FV, food vacuole; Apic, apicoplast; FLC, falcilysin; CLP,* Plasmodium* calpain; hCLP1, human calpain‐1; and PAP, serine proline aminopeptidase.

After egress from iRBCs, merozoites are vulnerable to detection by the immune system and are only viable for a few minutes during which they need to find and invade a new erythrocyte. Egress and invasion are therefore tightly coordinated and regulated processes with proteolysis playing major regulatory and effector roles. Proteases have long been known to be important in these pathways given that disruption of the parasitophorous vacuole and RBC membranes are required for parasite egress (Fig. 2A), general cysteine and serine protease inhibitors block egress, and the protein coat that covers the merozoite is proteolytically shed during invasion (Fig. 2B). A relatively recent proteomic study identified over 180 *Plasmodium* and host proteins that are cleaved during the last 6 h leading to parasite egress 26. These include not only RBC membrane and cytoskeletal proteins expected to be degraded during schizont rupture, but also proteins that are directly implicated in egress and invasion, and factors involved in protein trafficking and early parasite development within RBCs. While some of the key proteases involved in egress and invasion were identified more than a decade ago, only the recent development of conditional knock‐out (cKO) and knock‐down (cKD) systems is allowing us to understand the precise biological and molecular functions of these essential enzymes.

##### Proteases mediate parasite egress

Prior to egress, an increase in cGMP levels activates the cytosolic cGMP‐dependent protein kinase G, which triggers the secretion of proteins from apical organelles (exonemes and micronemes) into the parasitophorous vacuole (PV) and onto the merozoite surface 27 (Fig. 2A). Once secreted, the exonemal subtilisin‐like protease 1 (SUB1) processes several substrates that are important for egress and invasion 28. Among these are members of the serine repeat antigen (SERA) family, which in *P. falciparum* comprises nine proteins, each containing a papain‐fold catalytic domain. Only three are predicted to be active (SERA6–8) with the remaining six (SERA1–5 and 9) having a Ser instead of a catalytic Cys. SERA5 and SERA6 are the most abundant SERAs in blood stages and the only ones refractory to genetic KO 29, suggesting that they are important. SERA8 is mainly expressed in sporozoites and deletion of the *Plasmodium berghei* homologue prevents sporozoite egress from oocysts 30. Previous and on‐going work in the Blackman laboratory using the DiCre cKO approach has shown that whilst SERA5 is not essential, its cKO results in a premature egress phenotype that leads to a very significant decrease in invasion efficiency (M. J. Blackman, personal communication). Therefore, SERA5 is likely a pseudoprotease that regulates the timing of egress to coincide with the formation of fully mature and invasive merozoites.

Breakdown of the parasitophorous vacuole and RBC membranes (PVM and RBCM) allows the parasites to egress from iRBCs. Current work in the Blackman laboratory using the DiCre cKO system has shown that both SUB1 and SERA6 31 are essential: SUB1 is required for PVM breakdown while SERA6 is needed to disrupt the RBCM but not the PVM. Complementation of the SERA6 KO with different mutants strongly suggests that proteolytic activation of SERA6 by SUB1 is required for RBCM breakdown (M. J. Blackman, personal communication). It is not yet clear whether SUB1 or SERA6 are directly involved in the degradation of the RBC cystoskeleton. However, a recent study showed that processing of MSP1 (merozoite surface protein 1) by SUB1 is likely important for the destabilisation of the RBC cytoskeleton 32. In that study, Das *et al*. showed that processing of MSP1 triggers a conformational change that allows it to bind spectrin. Interestingly, parasites that endogenously express MSP1 mutants with inefficiently processed SUB1 cleavage sites egress significantly more slowly, suggesting that MSP1 cleavage might be a rate‐limiting step in parasite egress. This study is an excellent example of how the use of conditional systems can drastically change our understanding of parasite biology. MSP1 is the most abundant merozoite surface protein and provides a scaffold for the formation of the MSP1/6/7 complex, which was previously believed to be essential for RBC binding by released merozoites and invasion. However, conditional truncation of MSP1, to convert it to a soluble PV protein that is no longer GPI‐anchored to the merozoite surface, results in a significant egress defect but only a two‐fold decrease in parasite replication rate, thus showing that merozoites lacking MSP1 can invade RBCs. SUB1 is also expressed in liver stages where it has also been shown to play a role in egress using a cKO approach in *P. berghei* 33. However, in an independent study, cKO of PbSUB1 was shown to arrest schizont development and merozoite maturation within hepatocytes 34, indicating that SUB1 plays additional functions in liver stages compared to those in the erythrocytic cycle.

At the time of egress, human calpain 1 24 is activated at the RBCM, where it is thought to degrade components of the host cytoskeleton. Note that the cysteine protease dipeptidyl aminopeptidase 3 (DPAP3) was previously reported to be important to regulate parasite egress based on the observation that DPAP3 inhibitors block egress upstream of SUB1 activation 35. However, our current work using the DiCre cKO system provides very strong evidence that DPAP3 plays no significant role in egress but that it is important for efficient RBC invasion. Interestingly, DPAP2, which is only expressed in gametocytes 36, has been recently shown to reside in secretory organelles known as osmiophilic bodies, and its KO decreases gamete egress 37.

##### Merozoite maturases and sheddases ensure efficient RBC invasion

Proteases not only have an active role during invasion but they also ensure proper merozoite maturation before they escape the iRBC (Fig. 2B). The current model for RBC invasion involves initial recognition and attachment to the RBC surface, reorientation of the apical end towards the RBCM, active invagination and invasion of the RBCM using an actin/myosin motor, formation of the PV, and sealing of the PVM and RBCM 38, 39 (Fig. 2B). Many of the proteins involved in these events, including rhoptry, microneme, and surface proteins, are processed during merozoite maturation. However, with the exception of a few proteins (mainly SUB1 substrates), the significance of these cleavage events, or the proteases mediating them, are still unknown. Although MSP1 is not strictly required for invasion, SUB1 processing of MSP1 and MSP7 likely makes merozoites more invasive. SUB1 has also been shown to cleave several rhoptry proteins (RAP1, RhopH3 and RAMA) that are important for invasion, as well as PV (GBP130, Hsp70, EXP1) and erythrocyte membrane proteins (PfEMP2) 40. Finally, on‐going cKO studies on the aspartyl protease plasmepsin IX (PM‐IX) have shown that this protease acts as a maturase during merozoite formation, and that its activity is required for RBC invasion (D. Soldati‐Favre, personal communication).

During invasion, *Plasmodium* rhomboids and subtilisin‐like protease 2 (SUB2) shed the protein coat of the merozoite. Two rhomboids have been studied in detail in *P. falciparum*: PfROM4 localises at the parasite surface and has been shown to cleave the transmembrane domain of adhesin proteins that are important for parasite attachment to the RBC surface 41. In particular, PfROM4 has been shown to cleave EBA175, an adhesin important for the sialic acid‐dependent invasion pathway 42. Attempts to mutate EBA175's rhomboid cleavage site have been unsuccessful, suggesting that PfROM4‐mediated cleavage of EBA175 is important for RBC invasion. PfROM1 has a more canonical rhomboid specificity, localises to the apical end of merozoites 41, and is able to cleave AMA1 (apical membrane antigen 1) *in vitro* 41, 43. AMA1 is a transmembrane micronemal protein that is secreted onto the merozoite surface right before egress. It is one of the core components of the tight junction, a protein complex that links the parasite myosin/actin motor to the RBC surface. AMA1 bridges interactions between components of the motor and rhoptry‐derived proteins that are inserted into the RBCM after reorientation 44, 45. Movement of the tight junction from the apical to the posterior end of the merozoite, concomitant with the proteolytic cleavage of parasite‐RBCM interactions along the moving junction (i.e. AMA1 and MSP1 shedding) generates forward motion. KO studies in *P. berghei* suggest that PbROM1 is not essential during the erythrocytic cycle but that it plays an important role in liver stages, probably during hepatocyte invasion by sporozoites 43. Sporozoites need to be highly motile in order to cross the dermal barrier and to transverse and infect hepatocytes. In this case, the adhesin TRAP links the actin/myosin motor to the surface of host cells and its cleavage is required for motility. Rhomboids are likely responsible for TRAP cleavage since mutation of its putative rhomboid cleavage sites results in nonmotile sporozoites, which might explain the role of PbROM1 in liver infection 46. However, studies in *Plasmodium yoelii* have shown that PyROM1 is essential for proper PV formation 47 in hepatocytes rather than invasion, a phenotype that has not been confirmed in *P. berghei* 48. Although PbROM1 has been implicated in asexual replication, virulence, and oocyst formation, the results of these KO studies have not been consistent across different research groups 43, 48. In a systematic KO approach, Lin *et al*. showed that only half of the eight *P. berghei* rhomboids seem to be essential in blood stages (PbROM4, 6, 7 and 8), and PbROM3 is required to produce sporozoites. Nothing is known about the potential functions of ROM6–8, but ROM6 and ROM7 are predicted to localise to the mitochondria and apicoplast, respectively 48.

The other important sheddase for RBC invasion is SUB2, a transmembrane Ca^2+^‐dependent protease that is secreted from micronemes into the merozoite surface 49. SUB2 has been shown to shed the MSP1/6/7 complex as well as AMA1 50 and PTRAMP 51. SUB2 has been refractory to genetic deletion, suggesting an essential role. This has recently been confirmed by on‐going cKO studies in the Blackman laboratory (M. J. Blackman, personal communication). Interestingly, SUB2 was detected in the secretory osmiophilic bodies of gametocytes 37, and it is secreted from ookinetes during invasion of midgut epithelial cells, suggesting a role in sexual stages and in midgut wall transversal, respectively.

Note that plasmepsins VII and X (PM‐VII and PM‐X) are also expressed in ookinetes where they have been suggested to play a role in midgut transversal 52, but KO of PM‐VII in *P. berghei* has no effect in blood or insect stages 53. Also, while both PM‐VI and PM‐VIII are dispensable during the erythrocytic cycle, in *P. berghei* their KOs prevent sporozoite formation 54 and sporozoite motility 55, respectively, thus making them potential targets to block transmission.

Finally, although falcipain 1 (FP1) inhibitors seem to block invasion 56, KO of this protease in *P. falciparum* has no apparent effect on parasite development or invasion 57. Processing events taking place during egress and invasion might also be important for early parasite development within newly infected RBCs. For example, after shedding of MSP1 by SUB2, a 19 kDa GPI‐anchored fragment of MSP1 remains attached to the parasite membrane. This 19 kDa form persists in the food vacuole membrane, where it has been suggested to play a role in the biogenesis of this organelle 58.

#### Modifying the host cell

In order to survive within the RBC, the parasite needs to modify the RBC cytosol and membrane extensively to acquire metabolites, adhere to epithelial cells and evade the immune system. Around 10% of the parasite proteome is exported into the RBC through translocation of PV proteins across the PVM via the PTEX complex (*Plasmodium* translocon for exported proteins) 59. Most exported proteins contain a PEXEL (protein export element) motif downstream of the secretory signal peptide, which is processed by plasmepsin V (PM‐V) in the ER 60, 61. This cleavage exposes an N‐terminal sequence that is recognised by PTEX. PM‐V is therefore a very promising target since its inhibition will likely affect most extracellular functions.

Another mechanism by which the parasite releases proteins into the host cell is through secretion of rhoptry proteins at the time of invasion. These proteins have been shown to be implicated in PV formation, RBCM modification and nutrient uptake 62. Many rhoptry proteins contain a pro‐peptide downstream of the signal peptide that is proteolytically removed during merozoite maturation 63, 64. Although SUB1 has been shown to cleave some of these proteins *in vitro* 40, it is not known whether other proteases are also involved in this processing. Finally, modification of the RBCM allows iRBC to attach to epithelial cells. A recent study on a secreted proline aminopeptidase implicates this serine protease in RBC deformability and cytoadhesion 65. However, the role of proteases in these processes is poorly understood.

#### Eating the RBC content

To grow within RBCs, the parasite imports proteins from the host cytosol into the food vacuole where they are degraded by a panel of proteases into single amino acids (Fig. 2C). This pathway, known as the haemoglobin degradation pathway, provides amino acids for protein synthesis and liberates space within the RBC for the parasite to grow. The high level of proteolytic redundancy built into this pathway suggests that it is very important for parasite development, but also implies that individual proteases are not likely to be essential. Four aspartyl proteases, plasmepsins I‐IV (PM‐I, PM‐II, PM‐III and PM‐IV) 66, three papain‐like proteases, falcipains 2,2′ and 3 (FP2, FP2′ & FP3) 67, 68, 69 and the metalloprotease falcilysin 70 are responsible for the degradation of haemoglobin into smaller oligopeptides. Although PM‐I and PM‐II initiate this processing 71, 72, they are proteolytically activated by the falcipains 73. Falcipain inhibition leads to accumulation of undigested haemoglobin in the food vacuole resulting in an enlargement of this organelle 74. While each individual falcipain 74 could be genetically disrupted without significant effects on parasite growth, no double or triple falcipain KO has been reported, suggesting that as a family these proteases might be essential. (FP3 KO parasites were recently generated in the Goldberg laboratory with no apparent effect on parasite development, D. E. Goldberg, personal communication.) Similarly, individual KO of each of the four digestive plasmepsins has little effect in parasite replication, and parasites lacking all four (quadruple KO) are viable but grow significantly slower *in vitro* and are more sensitive to cysteine protease inhibitors 75, 76. This suggests that food vacuole plasmepsins might not be optimal drug targets, specially when compared to proteases that cannot be KO 77. That said, the importance/essentiality of a gene might be underestimated when evaluated in laboratory‐adapted strains growing under optimal conditions as opposed to an *in vivo* setting.

On the other hand, attempts to KO falcilysin have been unsuccessful 78, suggesting that it is important for parasite development. Interestingly, falcilysin has also been shown to localise to the apicoplast where it has been proposed to degrade apicoplast transit peptides after their removal by a signal peptidase 78. However, further studies are required to confirm this function and to determine which one of these two putative roles is more important for parasite development.

At the bottom of the haemoglobin degradation pathway is a panel of aminopeptidases: DPAP1 degrades oligopeptides into dipeptides 79, 80, and several food vacuole and cytosolic aminopeptidases (PfA‐M1, PfA‐M17, Pf‐APP, PfA‐M18) further cleave dipeptides and oligopeptides into single amino acids 81, 82. While PfA‐M18 KO parasites are viable, attempts to KO DPAP1, PfA‐M1, PfA‐M17 or Pf‐APP have been unsuccessful, suggesting that these proteases are important for parasite development. In addition, potent inhibitors against aminopeptidases 83, 84, 85, 86 and DPAP1 22 have been shown to have antiparasitic activity both *in vitro* and *in vivo*. However, genetic validation of these targets using conditional approaches is required to confirm the essentiality of these proteases and their biological functions.

It is important to mention that in *P. berghei* most proteases involved in the haemoglobin degradation pathway could be KO with the exception of the falcilysin and PfA‐M1 homologues 87. However, all KO lines except the PbBP2KO and PbA‐M18KO have lower replication rates than WT parasites. That said, *P. berghei* has a much smaller repertoire of digestive proteases having only one falcipain and one plasmepsin homologues (BP2 and PM‐IV, respectively), which suggests that the haemoglobin degradation pathway might not be equally important in all *Plasmodium* spp.

### Core biology proteolytic functions

Most studies on malaria proteases have focused on enzymes that perform parasite‐specific functions that often take place at a specific stage (Fig. 2C). From a pharmacological point of view, this implies that drugs might only be able to act within a short period of time during the parasite's life cycle. By contrast, proteases involved in core biological functions are more likely to be important throughout the parasite life cycle, including liver and insect stages. Also, these enzymes are generally constitutively active and therefore vulnerable to inhibition immediately after drug treatment, as opposed to proteases that are kept inactive as zymogens or bound to endogenous inhibitors until they are needed.

#### Protein trafficking

As mentioned above, the majority of proteins destined for the RBC cytosol or membrane are cleaved by PM‐V, which makes it a very promising target as its inhibition would block a variety of biological processes such as protein trafficking to the infected host cell surface, metabolite import, haemoglobin internalisation or Maurer's cleft formation. Similarly, targeting proteases involved in intracellular trafficking will likely disrupt parasite development at any stage. Most soluble proteins in the secretory pathway require co‐translational insertion of a hydrophobic N‐terminal signal peptide into the ER membrane, translocation of the polypeptide chain within the ER, and cleavage of the signal peptide. This is mediated by the signal peptidase complex, which recognises and cleaves the signal peptide in the lumen side of the membrane, and by a transmembrane aspartyl SPP, which cleaves it within the ER membrane. In *P. falciparum*, the two serine protease subunits of the signal peptidase complex (SP18 and SP21) have been reported to have proteolytic activity 88, 89, and the latter localises in the ER. In addition, PfSPP has been chemically validated as important for parasite development, and attempts to KO this gene have been unsuccessful 90. Importantly, potent PfSPP inhibitors have been shown to have low nanomolar potency against blood and liver stages and show little toxicity in human cell or animals, suggesting that effective antiparasitic PfSPP inhibitors could be safely developed 21.

Proteases involved in the trafficking of apicoplast or mitochondrial proteins might also be potential antimalarial targets. Nuclear‐encoded proteins destined for the apicoplast are directly transferred from the ER to the apicoplast via recognition of a bipartite transit peptide downstream of the N‐terminal signal peptide. In chloroplasts, this transit peptide is cleaved by the stromal processing peptidase 91. A homologue of this protease has been identified in *Plasmodium* spp. and is predicted to localise to the apicoplast 92. As mentioned before, falcilysin has been proposed to degrade this transit peptide 78. On the other hand, nuclear‐encoded mitochondrial proteins are translated in the cytosol and translocated through the inner and outer membranes via an N‐terminal bipartite presequence peptide. This peptide is usually cleaved by the mitochondrial processing peptidase (MPP), which releases proteins in the mitochondrial matrix. MPP is composed of two catalytic subunits (MPPα and MPPβ), both of which are encoded in *Plasmodium* genomes and predicted to localise to the mitochondria. Proteins destined to the intermembrane mitochondrial space also contain a hydrophobic sorting signal downstream of the MPP cleavage site that is inserted into the inner membrane 91. Serine signal peptidases belonging to the same family as SP21 are usually responsible for cleaving this sorting signal and releasing proteins into the intermembrane space. Although SP18 and SP21 are the only proteases from this family found in *Plasmodium* spp., bioinformatic analysis suggests that SP21 might also localise to the mitochondria. Alternatively, the predicted mitochondrial rhomboid, ROM6, might be able to cleave the sorting peptide within the inner membrane. Finally, the N‐terminus of matrix mitochondrial proteins is often further processed through removal of an octapeptide by the metalloprotease MIP (mitochondrial intermediate peptidase), whose homologue is also found in the *Plasmodium* genome and predicted to be mitochondrial 91.

#### Protein homeostasis

The vast majority of proteins within a cell are processed at least twice: first through removal of their N‐terminal methionine by Met‐aminopeptidases (MetAPs), and second, through their degradation by an ATP‐dependent proteolytic system such as the proteasome or in lysosomal organelles. Protein homeostasis is particularly important in *Plasmodium* species given its complex life cycle and the variety of distinct morphological stages, each requiring a special set of proteins. Indeed, 80% of genes expressed in the erythrocytic cycle are regulated in a cyclic manner 93. Proteases involved in protein quality control and timely degradation of unwanted proteins are likely to be essential for proper parasite development.

Five MetAPs have been identified so far in *Plasmodium* spp., and inhibitors of MetAP1b and MetAP2 have been shown to have antiparasitic activity both *in vitro* and *in vivo* 94, 95, 96. Removal of the N‐terminal methionine is also necessary for mitochondrial‐ or apicoplast‐encoded proteins. Interestingly, MetAP1c and MetAP2 are predicted to be trafficked to the apicoplast, while MetAP1a is likely a mitochondrial enzyme. However, to the best of my knowledge, no genetic studies have been performed to determine whether any single MetAP is essential, nor whether they perform redundant functions.

The proteasome/ubiquitination/deubiquitination system is not only crucial to regulate protein turnover and degrade misfolded proteins, but also to signal and regulate a variety of biological processes. The proteasome is predicted to be essential in all eukaryotes including *Plasmodium* spp. Recent studies on the structure of the *P. falciparum* proteasome combined with SAR studies have shown that there are significant differences in specificity between the human and malaria proteasomes that can be exploited to design safe *P. falciparum* proteasome inhibitors 20, 97.

In *Plasmodium*, ubiquitination has been linked to a variety of biological process such as the ER‐associated protein degradation (ERAD) pathway, response to oxidative stress, protein trafficking and drug resistance. Bioinformatic analysis predicts close to 30 deubiquitinating enzymes (DUBs) in *P. falciparum* including proteases predicted to cleave ubiquitin‐like modifiers such as SUMO, NEDD8, HUB1, URM1 or ATG8. Although some of these proteases are likely to be essential, understanding these pathways, which are likely to contain multiple redundant and/or overlapping functionalities, is very challenging, and the main reason why our knowledge of *Plasmodium* DUB‐like proteases is so sparse: UCH3 98 and UCH54 99 have both been shown to react with ubiquitin‐ and NEDD8‐based activity‐based probes (ABPs), suggesting that they have dual specificity. Mutations in UBP1 have been associated with decreased susceptibility to artemisinin but no further functional studies have been reported 100. A *Plasmodium* homologue of human USP14 was found to associate with the *Plasmodium* proteasome and has been shown to have DUB activity 101. Finally, SENP1 has desumoylation activity, a unique substrate specificity, and its inhibition seems to block parasite egress 18. However, this function needs to be confirmed genetically. The deubiquitination system remains one of the unexplored areas in malaria biology. From a therapeutic point of view, the use of broad‐spectrum DUB inhibitors might prove beneficial if drugs can be selectively targeted into iRBCs to minimise off‐target effects in the host.

Finally, site‐2 proteases (S2P), belonging to the M50 family of metalloproteases, are integral membrane proteins that cleave within the transmembrane domain of their substrate. S2Ps generally localise to the Golgi membrane where they have been shown to be involved in the unfolded protein response pathway. Two proteases belonging to the M50 family are present in *Plasmodium* spp. A recent study in *P. berghei* has shown that S2P is expressed throughout the parasite life cycle (liver, blood and insect stages), localises to the periphery of the nucleus, and its KO results in a significant impairment of parasite development in liver and asexual blood stages. It is not clear whether the second M50 protease plays a redundant or complementary function, nor whether inhibitors with dual specificity might have potent antimalarial activity 102.

#### Mitochondria and apicoplast biogenesis and maintenance

*Plasmodium* spp. have two organelles that originated from endosymbiotic events with ancestral prokaryotes: the mitochondrion and the apicoplast. Proteases are required to deliver proteins into these organelles, but also for their biogenesis, protein homeostasis and regulation of protein function. Therefore, mitochondrial and apicoplast proteases might be interesting antimalarial targets, especially if differences in specificity between human and *Plasmodium* homologues can be identified. Interestingly, a prokaryotic protease that was maintained in *Plasmodium* spp. but not in mammals is the bacterial proteasome system known as HslUV or ClpQY. This protein complex localises to the mitochondria 103 and is composed of 24 subunits arranged in a stack of four hexameric rings: two hexamers of proteolytic subunits (HslV or ClpQ) sandwiched between two hexamers of ATP‐dependent enzymes (HslV or ClpY) which unfold proteins and feed them into the central proteolytic cavity. In *P. falciparum* conditional overexpression of a dominant negative ClpQ mutant results in abnormal mitochondrial morphology, blocks organelle growth and division, and disrupts transcription of mitochondria‐encoded genes 104. Two other ATP‐dependent proteases are also important for the function of these organelles: the mitochondrial metalloprotease FtsH 105 and the apicoplast ATP‐dependent Ser protease system ClpAP 106. Inhibition of the latter prevents apicoplast growth 106.

Two likely essential rhomboids are predicted to localise to the mitochondria and apicoplast, called PfROM6 and PfROM7, respectively 48. In other eukaryotes, mitochondrial rhomboids, such as PARL in humans or Rbd1p/Pcp1p in yeast, have been shown to be involved in mitochondrial biogenesis, morphology, and functional regulation, as well as autophagy, apoptosis and cell signalling 107. PfROM6 and PfROM7 might therefore perform similar essential functions in the parasite mitochondria and apicoplast.

#### Cell cycle progression and programmed cell death

To date, *Plasmodium* calpain is the only protease that has been shown to localise to the nucleus and have a role in nuclear division 108, but its molecular function remains unknown. In terms of programmed cell death, similarities between apoptosis and drug‐mediated parasite death have been reported such as DNA fragmentation, chromatin condensation, loss of mitochondrial potential and presence of caspase‐like activity 109, 110. However, caspases are only found in metazoan organisms, and therefore, not present in *Plasmodium* spp. On the other hand, metacaspases are present in metazoans, plants, fungi and protozoa and have been shown to have a role in programmed cell death, which has led to the hypothesis that *Plasmodium* metacaspase‐1 (PfMCA‐1) might have a similar function 111. Interestingly, PfMCA‐1 has been shown to be active and able to complement the function of yeast metacaspase 110. However, KO of metacaspase in *P. berghei* has no effect in parasite development 112. Currently, there is no direct evidence that PfMCA‐1 induces parasite death, nor that *Plasmodium* spp. have evolved a programmed cell death pathway.

## Validation of proteases as antimalarial targets

The ubiquitous role of proteases in parasite biology and the proven use of protease‐targeting drugs to treat a variety of diseases make these enzymes very attractive candidates for antimalarial therapy. Medicines for Malaria Venture (MMV) recently published an updated target product and target compound profiles for new antimalarial therapies and drug candidates 113. Ideally, new therapies should be composed of a combination of 2–3 drugs, be safe, stable and cheap, and delivered using a regimen comprising no more than 1–3 oral doses. This treatment should be sufficient to reduce parasitaemia by more than 10^12^ within 72 h and reduce fevers within 24 h. It should also be clinically effective against drug‐resistant strains, prevent relapses due to hypnozoite activation, and block transmission by killing gametocytes, hepatic schizonts and/or the mosquito vector. Although these criteria are unlikely to be fulfilled by blocking any single target, to clinically validate a protease as a target, its inhibitor drug candidate needs to fulfil one or several of these requirements and also be tested in combination with other drugs. However, before investing too much effort in expensive drug development programs, I believe it is essential to validate potential protease targets at the genetic, biological, chemical and therapeutic level.

### Genetic validation

**Figure 3:**
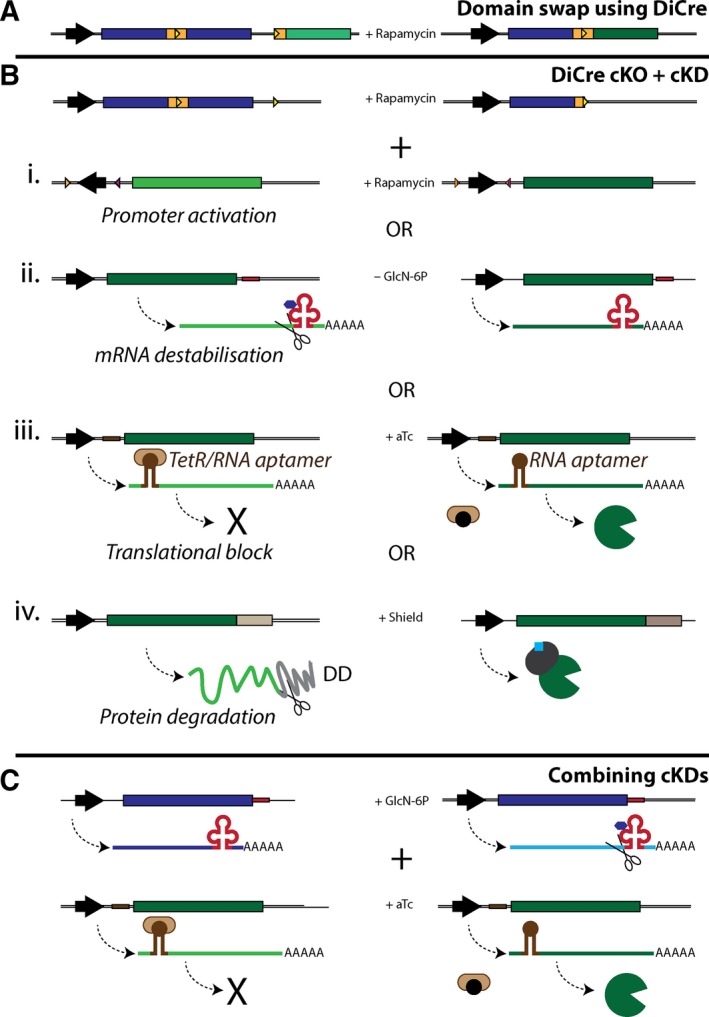
Conditional complementation strategies in *Plasmodium falciparum*. Targeted protease genes are depicted in blue and complementing copies in green. Promoters are represented by block arrows and LoxP sites by triangles. (A) Domain swap strategy using the DiCre system. Introduction of ‘silent’ LoxP sites within artificial introns (orange rectangles) allows replacement of the catalytic domain with a mutant version upon rapamycin treatment. (B) Combination of the DiCre cKO with cKD strategies. In all examples, rapamycin‐induced conditional truncation of the catalytic domain is coupled with the up‐regulation of the complementing protease (wild‐type or mutant): (i) LoxP sites facing opposite directions can be used to activate the promoter of the complementing protease by adding rapamycin. (ii) Introduction of the *glmS* ribozyme (in red) between the mRNA stop codon and its 3’‐UTR allows for post‐transcriptional regulation of the target of interest. Glucosamine‐6‐phosphate (GlcN‐6P, blue hexagon) activates the ribozyme resulting in mRNA degradation and downregulation of the complementation copy. Removal of GlcN‐6P after rapamycin treatment will turn‐on the complementing copy after excision of the protease of interest. (iii) RNA aptamers (dark brown) designed to bind the Tet repressor (light brown shape) can be inserted upstream or downstream of the mRNA ORF to prevent translation. Addition of anhydrotetracycline (aTc: black circles) results in dissociation of the Tet repressor, thus allowing translation of the complementation copy. (iv) Fusion of a degradation domain (grey) to the complementing protein leads to its proteasomal degradation. However, addition of shield (blue square) allows folding and stabilisation of the degradation domain, thus preventing degradation of the complementing protease. (C) Example of how the ribozyme and TetR/RNA‐aptamer strategies can be combined to achieve conditional knock down of the target of interest and upregulation of its complementation copy.

Genetic manipulation of *P. falciparum* has been challenging due to the A/T rich nature of its genome, the very low levels of homologous recombination and transfection efficiency, and the scarcity of selection markers. Traditional modification of the haploid asexual blood stages of *P. falciparum* by single or double crossover homologous recombination usually takes 3–6 months and involved transfection with circular DNA, selection of transfected parasites using a drug resistance marker, lengthy on/off drug cycles to enrich for integrant parasites and to encourage episome loss, and a final limiting dilution cloning step to obtain a population of genetically homogenous modified parasites. Failure to obtain KO lines using this method would only be suggestive of whether a gene is essential, since this inability might be due to inherent input plasmid stability problems or difficulty to target the selected locus. Also, genetic modifications that result in a fitness cost rather than parasite death will allow wild‐type parasites to outcompete the genetically modified ones. A further consideration is that the lengthy selection process might lead to evolutionary adaptation (e.g. by upregulation of genes with complementary functions), thus increasing the risk of mischaracterising the biological relevance of a target. Fortunately, over the last few years several cKO and cKD systems have become available providing much better tools to validate antimalarial targets genetically (Fig. 3) 9. Moreover, the use of CRISPR/Cas9 technologies and the introduction of additional positive and negative selection markers have provided the necessary tools to answer a variety of biological questions 114, 115.

In order to validate a protease as an antimalarial target, one must demonstrate that its proteolytic activity is essential for parasite replication. To avoid the above‐mentioned false positive or negative results, a conditional approach should be used. Although cKD systems can provide temporal, tuneable and reversible control of when to downregulate a target, they rarely achieve a 100% knock down. This might be problematic if low levels of protease activity are sufficient to perform its function. Furthermore, a partial block in parasite development might be due to insufficient knock down, or because the protease of interest is important but not essential. On the other hand, cKO approaches completely remove the gene of interest, thus providing a clearer picture about its essentiality. That said, phenotypic effects associated with a cKO might not be evident within a single cycle if sufficient protein or mRNA levels persist after removal of the gene of interest. Also, the versatility of cKD systems might be required to understand the role of a protease, especially if it performs multiple essential functions. For example, in the study by Suarez et al. 34, cKO of PbSUB1 in liver stages blocks merozoite formation, thus preventing the study of its downstream function in parasite egress.

The DiCre recombinase system is the only efficient cKO method currently available for *P. falciparum* 116, 117. DiCre recombines specific DNA sequences (LoxP sites) upon its activation with rapamycin. The Treeck laboratory further optimised this approach by introducing LoxP sites within artificial introns, thus permitting the introduction of ‘silent’ LoxP sites within the ORF of a gene, and allowing conditional removal or swapping of protein domains 118. Once a phenotype is observed, complementation studies with WT and a catalytically dead mutant are necessary to validate the essentiality of the proteolytic activity. Ideally, complementation should be performed by chromosomal genes under the control of the native promoter, and be conditional upon disruption of the native gene. This would minimise the chances of dominant positive or negative effects resulting from overexpression of WT or mutant proteases. The DiCre system is ideally suited to swap the catalytic domain of a protease with an identical WT or catalytically dead mutant (Fig. 3A). Alternatively, conditional approaches can be combined to turn‐on the complementing gene after cKO/cKD of the protein of interest (Fig. 3B,C). These conditional systems provide a great opportunity to characterise the phenotype associated with the loss of protease activity both at the cellular and biochemical level. These phenotypes can then be used as biomarkers of protease inhibition in drug development programs. A classic example of this is the use of the characteristic swollen food vacuole phenotype associated with the inhibition of falcipains 74.

### Biological and biochemical validation

Understanding the biological function of a protease is crucial in order to determine whether it will be a good antimalarial target. Knowing its role during the erythrocytic cycle might also provide clues as to whether this function is likely to be important in liver and insect stages. For example, proteases involved in schizont rupture are likely to be important in other egress events such as merozoite release from hepatocytes, gametes from iRBCs, or sporozoites from oocysts (Fig. 1). Similarly, proteases that play a role in RBC invasion might also be important for gliding motility, midgut transversal or hepatocyte invasion.

It is also important to keep in mind that *in vitro* conditions are rather artificial compared to those encountered *in vivo*. Many essential aspects of parasite biology important for pathogenesis are inconsequential in cultures, such as attachment to epithelial cells, sequestration, or evasion of the innate and adaptive immune responses. However, proteases involved in these processes are likely to be valuable targets. Similarly, while *in vitro* cultures are generally maintained under static conditions, in the human host iRBCs are either in rapid motion in the circulation or attached to epithelial cells, and continuously experience shear forces from the blood stream. Upon egress, merozoites are released into the laminar blood flow where they need to bind an invade RBCs. How this physical environment impacts the significance of proteases involved in these processes is an important question to take into consideration. Finally, parasites are generally grown *in vitro* under optimal media conditions. Therefore, they might be less reliant on efficient nutrient import mechanisms or on specific metabolic pathways than *in vivo*. The importance of proteases directly or indirectly involved in parasite metabolism will therefore likely be dependent on the environment. *P. falciparum* can obtain all natural amino acids except isoleucine from the haemoglobin degradation pathway 8. However, parasites are less likely to rely on this pathway if an abundance of amino acids are present in the media. For example, it has been suggested that the combined activities of DPAP1 and PfAPP (proline aminopeptidase) during haemoglobin degradation are required in order to salvage enough proline for protein synthesis 80.

Understanding the role of a specific protease within its proteolytic pathway will also determine whether it is a viable target. Does the protease of interest have a redundant function, as is the case of falcipains and plasmepsins in the food vacuole 8? Is it part of a proteolytic cascade, and does it perform a signalling or effector role? Is its proteolytic activity a rate‐limiting step in a biological process, such as cleavage of MSP1 by SUB1 during egress? 32 These questions are also important to understand the pharmacological requirements of a protease inhibitor and to prioritise targets within a proteolytic pathway.

Finally, the biological function of a protease can only be understood through the identification of its natural substrate(s) and the validation of these cleavage events as being important for parasite development. Global proteomic methods specifically developed to identify protease substrates such as TAILS or COFRADIC 119 can be used in combination with cKO/cKD systems or specific inhibitors. Validated substrates can then be used as biochemical markers to confirm that sufficient level of protease inhibition is achieved to exact a downstream effect. This is especially important because some proteases require a very high level of inhibition before starting seeing downstream effects.

### Chemical validation

Chemical validation of a target has to demonstrate that its inhibition by a small molecule at biologically relevant concentrations is sufficient to generate a phenotype. This requires the development of assays that directly measure protease inhibition under living conditions (within cells and/or *in vivo*), but also to provide sufficient evidence to show that lead inhibitors are not acting through off‐target or general cytotoxic effects.

#### Chemical tools to confirm protease inhibition in live parasites

The most commonly used chemical tools to measure protease activity are synthetic peptide‐based substrates that become fluorescent upon proteolytic cleavage, and ABPs, which covalently tag the active site of a protease. The advantage of substrates over ABPs is that they can measure substrate turnover continuously and therefore detect changes in protease activity upon addition of an inhibitor or stimulation of a proteolytic pathway. Also, substrates are generally more sensitive than ABPs due to the accumulation of signal over time, and they can be easily used in high‐throughput screening assays. However, it has been very difficult to develop highly specific substrates able to measure the activity of a single protease in living cells. The recent incorporation of non‐natural amino acids in positional scanning substrate libraries (HyCoSuL, hybrid combinatorial substrate libraries, and CoSeSuL, counter selection substrate libraries) has allowed to expand the chemical diversity of peptide‐based substrates and provides a very successful approach to identify differences in specificity between proteases 120. Substrates containing non‐natural amino acids tend to be more potent and specific and could be used to measure the activity of a single protease in live parasites. Here, the use of cKO/cKD lines provides a perfect opportunity to validate such substrates as highly specific.

**Figure 4:**
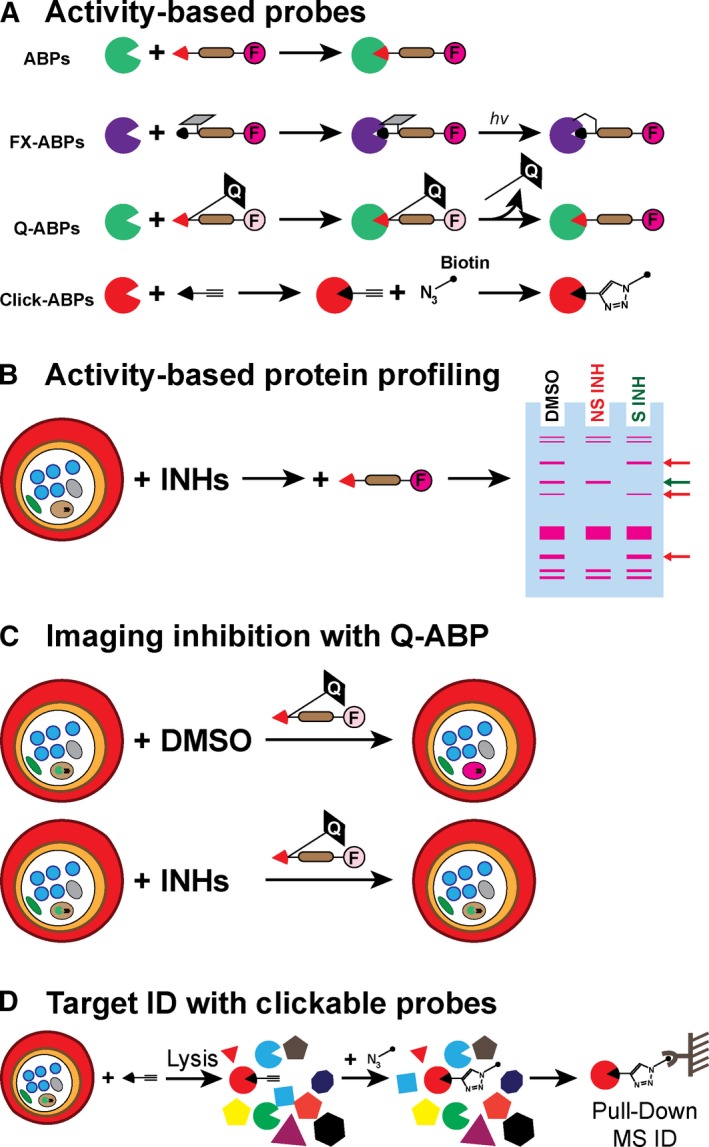
Activity‐based probes as tools to study protease function. (A) ABPs are composed of: an electrophile (red triangle) that covalently modifies the catalytic nucleophilic residue of a protease; a recognition element (brown shape) that targets the probe towards specific proteases; and a tag, usually a fluorophore (pink circle), that allows for visualisation of labelled proteases. In the case of Asp or metalloproteases, which lack a nucleophilic residue, covalent interactions between the protease and the ABP can be obtained by using a photo‐crosslinker (represented in grey, FX‐ABPs). Quenched‐ABPs (Q‐ABPs) contain a quencher (black shape) within the leaving group of the electrophilic warhead that renders the probe nonfluorescent. Covalent modification of the protease results in the release of the quencher and an increase in fluorescent signal. Clickable ABPs are small molecule inhibitors containing a clickable handle (usually an alkyne group) that can be used to couple different tags to the probe after treatment of intact cells. (B) Broad‐spectrum ABPs use the conserved mechanism of an enzyme family to covalently modify all members of a protease family. These can be separated by SDS/PAGE and their labelling visualised as fluorescent bands. Specific inhibition of a protease (S INH) will result in the loss of signal for a single specific band (green arrow). Nonspecific inhibitors (NS INH) will block labelling of multiple bands (red arrows). (C) Quenched‐ABPs can be used to visualise protease activity in living cells since the probe only becomes fluorescent after binding to the protease of interest. This results in a localised increased of fluorescence signal within the subcellular compartment where the active protease resides. These probes can be used to measure real‐time target activation or inhibition. (D) Addition of an alkyne group to a lead inhibitor usually does not alter its biological activity. These clickable probes can be used to confirm target inhibition and identify potential off‐targets. After pretreatment of living cells with the clickable probe, cells are lysed, biotin linked to the alkyne group via click chemistry, and the targets of the compounds pulled down and identified by MS.

Activity‐based probes are excellent tools to measure protease activity under living conditions (Fig. 4A). These small molecules use the catalytic mechanism of an enzyme to modify its catalytic nucleophilic residue (Cys, Ser or Thr) covalently. A peptidic (or peptidomimetic) sequence targets the probe towards specific proteases, and a tag, usually a fluorophore or biotin, allows for visualisation of the labelled proteases on an SDS/PAGE gel 121. In the case of metallo and aspartyl proteases, which lack a nucleophilic residue, ABPs have been developed by attaching a tag and a photocrosslinker to the scaffold of a potent inhibitor. This allows covalently linkage of the probe to the protease of interest. Because ABPs bind into the active site of proteases, they are excellent tools to identify competitive inhibitors. In the context of malaria, ABPs have been used to study cysteine (FPs 56, DPAPs 35, UCH 98, 99, SENP 18, human calpain 1 24), serine (SUB1 35, ClpAP 106), metallo (aminopeptidases 83) and threonine (proteasome 25) proteases, and to determine the potency and specificity of inhibitors in live parasites both *in vitro* and *in vivo* 22. One of the advantages of using ABPs rather than fluorogenic substrates is that they do not need to be highly specific since the protease of interest can be separate from other labelled proteases by SDS/PAGE. In fact, broad‐spectrum ABPs designed to label all members of a protease family are excellent tools to determine the specific of an inhibitor against each member of that family (Fig. 4B) 121. This is particularly important because protease inhibitors often cross‐react with other proteases within the same family. On the other hand, if a sufficiently specific ABP can be designed, a quencher can be added to the leaving group of an electrophilic warhead such that the probe will only become fluorescent upon reaction with the protease of interest. These quenched‐ABPs have been used for imaging protease activation or inhibition by live microscopy (Fig. 4C) 121.

Another chemical strategy to determine whether inhibitors act on target is the use of structure–activity relationship (SAR) series. Using one of the assays described above, target inhibition in live parasites can be directly correlated with the expected phenotype in a dose‐dependent manner. If this correlation holds true for different classes of inhibitors, it provides stronger evidence that compounds are acting on target. It is important to emphasise that any inhibitor development program should include the synthesis of negative control compounds in order to confirm that lead inhibitors are acting on target. These should be designed with minimal alteration to the structure of lead compounds: for example, a diastereomer of the inhibitor, a nonreactive version of a covalent inhibitor, or the addition of a sterically clashing methyl group.

Finally, a decrease or increase in inhibitor sensitivity upon overexpression or knock down of the target of interest is a good indication that the compound is acting on target. This method was used to demonstrate the on‐target effect of a PM‐V inhibitor by showing that parasites become more or less sensitive to the compound upon cKD or overexpression of PM‐V, respectively 122.

#### Phenotypic assays to confirm target inhibition

Once a phenotype associated with the loss of a particular protease activity has been characterised, an assay can be developed to quantify this phenotype and correlate it with target inhibition in live parasites. If inhibition of a protease is expected to arrest parasite development at a specific stage, this should be confirmed microscopically and/or using FACS‐based assays, which allow high‐throughput quantification of different intraerythrocytic stages. More specific microscopy‐based assays can also be developed if depletion of the protease of interest results in clear morphological changes, such as enlargement of the food vacuole upon falcipain inhibition, or by using fluorescent reporters. For example, episomal expression of a GFP‐tagged truncated and inactive form of ClpP was used to visualise the effect of ClpP inhibition in apicoplast morphology 106. Similarly, mitotracker was used to monitor mitochondrial disruption upon expression of a dominant negative HslV mutant 104.

If substrate cleavage mediates a change in its localisation, biochemical fractionation of infected RBCs can be used to monitor this change by western blot or ELISA assays. For example, inhibition of merozoite sheddases should decrease the amount of surface proteins released into the media supernatant after an invasion assay. Expression of fluorescent reporters can also be used to confirm protease inhibition in live parasites. For example, fusion of GFP to a PEXEL‐containing exported protein has been used to confirm PM‐V inhibition by live microscopy, which results in accumulation of GFP signal in the parasitophorous vacuole 123. Overall, the recent advances on high‐content and FACS‐based screening technology coupled with the use of fluorescent chemical reporters (substrates, ABPs, lyso/mitotracker) or protein markers should allow us to design high‐throughput protease‐specific cell‐based assays to facilitate target‐based drug development efforts.

#### Methods to identify targets and off‐targets of protease inhibitors

A common strategy to identify the targets of antimalarial compounds is to culture parasites at increasing compound concentrations to force the emergence of resistance. Full‐genome sequencing allows the identification of point mutations responsible for the resistance phenotype. These SNPs often cluster to genes encoding the targets of the compound 124. This approach can also be used to confirm that protease inhibitors act on target. However, there can be a variety of mechanisms by which parasites become resistant, such as pumping the inhibitor out, enzymatically degrading it, or through up or downregulation of alternative compensatory pathways. It is therefore important to confirm biochemically and genetically that the identified mutations alter the affinity of the inhibitor towards the target of interest.

A more direct approach to identify the targets of a compound is by using chemical proteomic methods. As mentioned above, activity‐based protein profiling provides a useful tool to determine the specificity of compounds against all members of an enzyme family (Fig. 4B). However, it provides little information about off‐target effects in unrelated proteins. Addition of a small alkyne group to the structure of an inhibitor usually does not alter its biological activity and can be used to pull down its targets after pretreatment of intact cells with compound, cell lysis and attachment of biotin to the alkyne group via click chemistry 125 (Fig. 4D). Alternatively, new unbiased quantitative proteomic methods, such as DARTS (drug affinity responsive target stability), CETSA (cellular thermal shift assay) or TPP (thermal proteome profiling), measure the increase in protein stability associated with compound binding and can be applied to confirm target inhibition and identify off‐targets 126.

### Therapeutic validation

Once a target has been genetically, biologically and chemically validated, the tools used to obtain this information can be repurposed to determine the pharmacological profile a protease inhibitor needs to meet in order to act as an efficient drug. A thorough *in vitro* understanding of these pharmacological requirements is necessary not only to determine whether a protease will be a viable target, but also to help develop inhibitors with well‐tuned pharmacology.

#### When, where and for how long is a protease active?

Proteases are generally expressed as zymogens that need to be activated at a specific time and place to perform their biological function. Protease activity is also tightly regulated through the timely expression and targeted degradation of endogenous inhibitors. Understanding when and where within the parasite life cycle a protease becomes active, and for how long its activity is required, will help define the desired pharmacological profile of inhibitors. The use of ABPs can be instrumental in answering these questions since these chemical tools can differentiate between the active and inactive forms of a protease. Inhibitor treatment at different life stages and for different periods of time can help define for how long a protease needs to be inhibited to block parasite replication, and whether parasites are more sensitive to protease inhibition at a specific stage. For example, food vacuole proteases are constantly being turned over and therefore will require inhibitors able to cross four membranes and sustain target inhibition for several hours in a highly acidic and oxidative environment 22. On the other hand, proteases that are activated for a very short period of time will likely require fast‐acting inhibitors. However, it is important to differentiate between how fast a protease performs its proteolytic function, and the window of opportunity during which its active site is targetable by a small molecule. For example, proteases whose function is regulated through compartmentalisation of mature enzyme in specific organelles, such as SUB1 in the exonemes or SUB2 in micronemes, could be targetable within their respective organelles several hours before they perform a rapid proteolytic function, i.e. SUB1 mediates egress in less than 30 min after exoneme secretion, and SUB2 sheds the merozoite protein coat in ~ 1 min during RBC invasion. Finally, understanding where a protease is active might help to incorporate specific chemical properties in the inhibitor structures. For example, compounds designed to have lysotropic effect will be ideal to target proteases residing in acidic organelles.

#### Mechanism of action and drug resistance

The development of antimalarial drugs needs always to be evaluated in the context of drug resistance. To avoid its emergence, drug development efforts have been mainly focused on identifying fast‐killing drugs as opposed to compounds that delay or halt parasite development. Specific assays that discriminate between cidal and static effects of compounds 127, to determine how fast a small molecule kill the parasite (time of killing assay) 128, 129, or to measure how easily parasites become resistant to a drug 130, 131, have been developed and should be applied to selective protease inhibitors. These assays can be used not only to validate proteases as therapeutic targets, but also to prioritise inhibitor scaffolds towards fast‐acting cidal compounds with low propensity to induce drug resistance. Finally, it is also important to show that current drug‐resistant strains, isolated from field isolates or generated *in vitro*, have similar sensitivity to lead protease inhibitors.

An effective strategy to prevent the emergence of resistance is the use of combination therapy. In the context of protease inhibitors, it is important to evaluate what would be a suitable antimalarial drug partner. For example, the mechanism of action of artemisinin involves the Fe^2+^‐mediated activation of an internal peroxide that generates toxic carbon‐centred radicals and increases oxidative stress. Because Fe^2+^ originates from the degradation of haemoglobin, falcipain inhibitors have antagonistic effects with artemisinin 132. On the other hand, inhibition of the proteasome makes the parasite more vulnerable to oxidative stress, resulting in synergistic effects with artemisinin 20. Therefore, isobologram studies between lead protease inhibitors and current and/or upcoming antimalarial drugs should be performed to identify synergistic partners 133.

#### New *in vitro* and *in vivo* models to evaluate potential antimalarial drugs

In recent years, industry and academic groups have invested considerable efforts into developing medium‐ to high‐throughput cell‐based assays to screen compounds that might block transmission. These include assays to monitor gametocyte development and egress, liver stage development, including hypnozoite formation and reactivation, and parasite transmission assays 10. These can be used to test whether specific protease inhibitors are effective in these parasite stages. Similarly, there has been a significant increase in the number of *in vivo* models available to evaluate the potential of new antimalarial drug. In addition to the most commonly used *P*. *berghei* and *P*. *yoelii* murine models, the *Plasmodium chabaudi* model has been robustly validated as a more accurate immunological model of malaria which can be used to study the acute and chronic phases of infection 12. The use of immuno‐compromised mice (SCID) has also allowed researchers to study *P. falciparum* liver and blood stages within a living host by grafting human hepatocytes and erythrocytes into mice. Although these models do not properly reflect the immune and inflammatory responses to parasite infection, they are valuable tools to assess the efficacy of small molecules in an *in vivo* system. In theory, cKO/cKD *P. falciparum* lines could be used in the SCID model to test the essentiality of a target within a living host. Finally, *Plasmodium knowlesi* is more closely related to *Plasmodium vivax*,* Plasmodium malariae* or *Plasmodium ovale* than *P*. *falciparum*, and its recent adaptation to grow in human erythrocytes 134 makes it an excellent *in vitro* model to predict the efficacy of lead inhibitors against these other malaria‐causing species.

## Conclusion

Over the last two decades, the rising interest in tackling neglected infectious diseases has resulted in a substantial increase of funds to study these pathogens, a stronger involvement of the pharma industry, and the creation of nonprofit and public–private partnership institutions to facilitate drug development programs. In the area of malaria, this resulted in a first wave of pharmacology‐based drug development programs including not only large high‐throughput phenotypic screening campaigns, but also development of malaria‐specific pharmacological assays and screening methodology to monitor all parasite stages. In the last few years, there has been a resurgent interest in target‐based approaches 135, in part due to the identification of targets from lead antimalarial compounds 124, 136, but more importantly, due to our much‐improved ability to modify *Plasmodium* spp genetically. In particular, the development of conditional genetic approaches in *P. falciparum* is allowing us for the first time to validate essential genes and study their molecular functions. I anticipate that these advances will soon result in a surge of target‐based antimalarial drug development programs.

In parallel, over the last 30 years there has been a dramatic shift in our understanding of protease function, from being purely degradative enzymes, to the realisation that proteases are, similarly to protein kinases, tightly regulated enzymes playing signalling and effector roles in most biological processes. The ubiquitous role of proteases in human diseases has made them one of the preferred enzyme families for target‐based drug development and has resulted in the implementation of a variety of chemical and proteomic methods to develop specific inhibitors, understand protease regulation and identify their natural substrates.

Proteases play crucial roles at all stages of parasite development and are therefore potential antimalarial targets. Importantly, only one‐third of predicted malaria proteases have been studied so far, and only a few of these have been characterised in any detail (Table 1). Thus, the potential of proteases as antimalarial targets is likely underestimated at the moment. The current advances in malaria genetics and chemical biology provide a unique opportunity to understand the biological function of *Plasmodium* proteases and achieve a robust genetic, biological and chemical validation of these targets. Finally, the variety of malaria‐focused pharmacological assays and *in vivo* models developed over the last decade will greatly facilitate the therapeutic validation of proteases as viable antimalarial targets.
